# Effectiveness of non-invasive brain stimulation on depressive symptoms targeting prefrontal cortex in functional magnetic resonance imaging studies: a combined systematic review and meta-analysis

**DOI:** 10.1093/psyrad/kkae025

**Published:** 2024-11-05

**Authors:** Yao Xiao, Shuai Dong, Chunyu Pan, Huiling Guo, Lili Tang, Xizhe Zhang, Fei Wang

**Affiliations:** Early Intervention Unit, Department of Psychiatry, Affiliated Nanjing Brain Hospital, Nanjing Medical University, Nanjing 210029, China; Functional Brain Imaging Institute of Nanjing Medical University, Nanjing 210029, China; Early Intervention Unit, Department of Psychiatry, Affiliated Nanjing Brain Hospital, Nanjing Medical University, Nanjing 210029, China; Functional Brain Imaging Institute of Nanjing Medical University, Nanjing 210029, China; School of Biomedical Engineering and Informatics, Nanjing Medical University, Nanjing 211166, China; School of Computer Science and Engineering, Northeastern University, Shenyang 110167, China; Early Intervention Unit, Department of Psychiatry, Affiliated Nanjing Brain Hospital, Nanjing Medical University, Nanjing 210029, China; Functional Brain Imaging Institute of Nanjing Medical University, Nanjing 210029, China; Early Intervention Unit, Department of Psychiatry, Affiliated Nanjing Brain Hospital, Nanjing Medical University, Nanjing 210029, China; Functional Brain Imaging Institute of Nanjing Medical University, Nanjing 210029, China; Early Intervention Unit, Department of Psychiatry, Affiliated Nanjing Brain Hospital, Nanjing Medical University, Nanjing 210029, China; School of Biomedical Engineering and Informatics, Nanjing Medical University, Nanjing 211166, China; Early Intervention Unit, Department of Psychiatry, Affiliated Nanjing Brain Hospital, Nanjing Medical University, Nanjing 210029, China; Functional Brain Imaging Institute of Nanjing Medical University, Nanjing 210029, China

**Keywords:** non-invasive brain stimulation, prefrontal cortex, depressive symptom, functional magnetic resonance imaging, activation likelihood estimation meta-analysis, meta-regression

## Abstract

The prefrontal cortex (PFC) is a critical non-invasive brain stimulation (NIBS) target for treating depression. However, the alterations of brain activations post-intervention remain inconsistent and the clinical moderators that could improve symptomatic effectiveness are unclear. The study aim was to systematically review the effectiveness of NIBS on depressive symptoms targeting PFC in functional magnetic resonance imaging (fMRI) studies. In our study, we delivered a combined activation likelihood estimation (ALE) meta-analysis and meta-regression. Until November 2020, three databases (PubMed, Web of Science, EMBASE) were searched and 14 studies with a total sample size of 584 were included in the ALE meta-analysis; after NIBS, four clusters in left cerebrum revealed significant activation while two clusters in right cerebrum revealed significant deactivation (*P* < 0.001, cluster size >150 mm^3^). Eleven studies were statistically reanalyzed for depressive symptoms pre–post active-NIBS and the pooled effect size was very large [(*d* = 1.82, 95%CI (1.23, 2.40)]; significant moderators causing substantial heterogeneity (Chi squared = 75.25, *P* < 0.01; *I*^2^ = 87%) were detected through subgroup analysis and univariate meta-regression. Multivariate meta-regression was then conducted accordingly and the model suggested good fitness (*Q* = 42.32, *P* < 0.01). In all, NIBS targeting PFC balanced three core depressive-related neurocognitive networks (the salience network, the default mode network, and the central executive network); the striatum played a central role and might serve as a candidate treatment biomarker; gender difference, treatment-resistant condition, comorbidity, treatment duration, and localization all contributed to moderating depressive symptoms during NIBS. More high-quality, multi-center randomized controlled trails delivering personalized NIBS are needed for clinical practice in the future.

## Introduction

Depression is a common mental disorder worldwide with a high prevalence of 15–18% (Malhi and Mann, 2018). Nevertheless, effectiveness of traditional pharmacological treatment is suboptimal and its precise mechanism remains unknown (Willner *et al*., 2013). In recent years, progresses have been made in developing and optimizing novel somatic treatments (Nemeroff, 2020). Unlike invasive deep brain stimulation or non-invasive yet convulsive electroconvulsive therapy, non-invasive brain stimulation (NIBS) including transcranial magnetic stimulation (TMS) and transcranial electrical stimulation (tES) are delivered through non-implantable devices and patients remain conscious during intervention with relatively few safety concerns. NIBS has been increasingly applied to clinical practice in recent years (Dandekar *et al*., 2018; Pinna *et al*., 2018; Brunoni *et al*., 2019). Based on the parameters, TMS could be divided into high-frequency repetitive TMS (≥5 Hz), low-frequency repetitive TMS (≤1 Hz), and theta-burst stimulation (TBS) while tES are classified into transcranial direct current (tDCS), transcranial alternating current, and transcranial random noise stimulation, and repetitive TMS (rTMS) as well as tDCS are among the most commonly used in the treatment of depression (Klomjai *et al*., 2015; Ghobadi-Azbari *et al*., 2021).

The prefrontal cortex (PFC) is generally known as a critical brain region accounting for numerous advanced brain activities such as working memory, cognition, and emotional regulation (Carlén, 2017). Neural deficits have been detected in the PFC of patients with depression, coupled with significant normalizations after antidepressant treatment (Lai, 2021). This provided strong basis for delivering NIBS targeting PFC. To date, the dorsolateral PFC (dlPFC) is mostly acknowledged for relieving depressive symptoms and has been approved by the United States Food and Drug Administration (FDA) as an effective rTMS target for treatment-resistant disorder (TRD) (Perera *et al*., 2016; Lefaucheur *et al*., 2014; Lefaucheur *et al*., 2020). However, growing evidence also suggests that dorsal medial PFC (dmPFC) has the potential to be a novel neurostimulation target region, the intervention of which was shown to be at least as effective as dlPFC (Downar *et al*., 2014; Bakker *et al*., 2015). Furthermore, exploratory tDCS studies targeting both ventromedial and ventrolateral PFC among healthy participants revealed significantly regulated emotional processing, thus indicating the substantial therapeutic use of ventral PFC stimulation in various mental disorders (Lefaucheur *et al*., 2017; Vergallito *et al*., 2018; Junghofer *et al*., 2017). In all, including but not limited to dlPFC, the whole prefrontal region is demonstrated as an important NIBS target region for relieving depressive symptoms, yet its effectiveness requires further exploration.

Functional magnetic resonance imaging (fMRI) is demonstrated as an objective approach for mapping depression beyond symptoms. A number of NIBS studies targeting PFC have employed regional (e.g. amplitude of low-frequency fluctuation) as well as connective (e.g. functional connectivity, FC) indicators to assess brain activity post-intervention, and normalization of dysfunctional brain activations suggest that neuroimaging alterations could be regarded as a potential biomarker for appraising treatment effectiveness (Esmaeilpour *et al*., 2020; Jog *et al*., 2019; Dichter *et al*., 2015; Chrysikou *et al*., 2019; Iwabuchi *et al*., 2019). However, the existing results remain inconsistent, and the sample sizes of neuroimaging studies are relatively small, both of which urge the need to conduct an activation likelihood estimation (ALE) meta-analysis so that a unified conclusion concerning changes of brain activations after NIBS could be made, through which we might grasp a better understanding of NIBS's mechanism and deliver more precise interventions accordingly (Eickhoff *et al*., 2012; Turkeltaub *et al*., 2002). On the other hand, although symptom-based treatment efficacy is generally accepted, various factors such as participants’ characteristics, seriousness of depression, and intervention parameters all contribute to the heterogeneity between studies; clinical markers were adopted to predict response to NIBS treatment, yet the results suffer a lack of repeatability (Kar, 2019; Kraus *et al*., 2020). Hence, a multi-center, large-scale meta-regression could help optimize and personalize intervention protocols, which is of great clinical value.

To date, no combined ALE meta-analysis and meta-regression concerning effectiveness of NIBS on depressive symptoms targeting PFC in fMRI studies has been made. Deng *et al*. systematically evaluated the effect of TMS treatment on depression (Deng *et al*., 2020); compared to our study, however, it was lacking in tES interventions. Another meta-analysis systematically reviewed efficacy of NIBS on cognitive function in brain disorders (Begemann *et al*., 2020), but no neuroimaging results were included. In all, the major objectives of our study are: (i) to systematically review the present neuroimaging fMRI studies that aim at relieving depressive symptoms through NIBS and investigate the alterations of brain activity post-intervention targeting PFC through ALE meta-analysis; and (ii) to detect clinical factors that could significantly improve depressive symptoms and build a model of clinical use through meta-regression.

## Material and methods

### Protocol and Registration

This systematic review and meta-analysis followed the Preferred Reporting Items for Systematic Reviews and Meta-Analyses (PRISMA) guideline (Moher *et al*., 2009); the study was under registration on PROSPERO (ID: 238 542).

### Eligibility Criteria

The inclusion and exclusion criteria were as follows:

Inclusion criteria:

(i) Randomized controlled trails (RCTs), case control studies, and cohort studies
(ii) Task or resting-state fMRI studies
(iii) Participants were diagnosed with major depressive disorder (MDD) or appeared to have depressive symptoms
(iv) NIBS was delivered targeting PFC
(v) fMRI scans covering the whole brain were conducted after NIBS
(vi) Coordinates of peak voxels post-intervention were reported.

Exclusion criteria:

(i) Protocols, reviews, or meta-analyses
(ii) Voxel-based morphometry, diffusion tensor imaging, positron emission tomography, and magnetic resonance spectroscopy studies
(iii) Participants were comorbid with brain injuries, strokes, Parkinson's diseases, and other neurological diseases.

### Search Strategy

A preliminary search in PROSPERO and the Cochrane Library was conducted to avoid duplicate study and no duplication was found. Key words were composed of four parts: depression, fMRI, NIBS, and PFC (Supplementary Material Table S1). Notably, PFC subregions containing the dlPFC, dmPFC, ventrolateral PFC, and ventromedial PFC were all included as search terms; besides, mesh terms of all key words were used to increase sensitivity. Two authors (Y.X., C.Y.P.) retrieved the listed key words in PubMed, EMBASE, and Web of Science for eligible studies published until November 2020. One author (H.L.G.) manually identified included studies in relevant meta-analyses to make sure no study was left out. Then two authors (Y.X., C.Y.P.) separately eliminated studies according to exclusion criteria; for studies with disagreements, a third author (H.L.G.) was supposed to make final decisions. When studies did not report complete data, requesting e-mails were sent to the corresponding authors; meanwhile, results of all studies registered with the same NCT numbers were also looked over for useful information by one author (H.L.G.).

### Risk of Bias

For all the eligible studies, one author (Y.X.) evaluated RCTs’ risk of bias (RoB) according to the Cochrane Collaboration's tool; RoB was composed of seven domains, namely sequence generation, allocation concealment, blinding of participants and personnel, blinding of outcome assessment, incomplete outcome data, selective outcome reporting and other bias; levels of RoB were categorized into low, unclear, or high (Higgins *et al*., 2019). One author (C.Y.P.) evaluated RoB of case control studies and cohort studies through the Cochrane-recommended Newcastle-Ottawa Quality Assessment Scale; for case control studies, three categories including selection, comparability, and exposure were suggested as main indicators; for cohort studies, three categories including selection, comparability, and outcome were suggested as main indicators. Both scales contained a total score of nine points (http://www.ohri.ca/programs/clinical_epidemiology/oxford.asp).

### Quality of Evidence

Since there was no standard criteria for assessing quality of imaging studies, we referred to the quality assessment protocol of another coordinate-based meta analysis; one author (H.L.G.) accordingly evaluated studies included in our meta-analysis (Deng *et al*., 2020).

### Data Extraction

Two authors (L.L.T., S.D.) collected data of the following items, as recommended by the standard checklist from the Cochrane Handbook (Higgins *et al*., 2019):

Source: author, country, year of publication;

Methods: type of fMRI, type of NIBS, type of comparison, type of control group, intervention target region, localization method, intervention parameters, total sessions, treatment duration, evaluation duration, combined-intervention, RoB;

Participants: age, gender (proportion of female), comorbidity, treatment-resistant condition, medication;

Results: overall sample size, sample size of two comparisons, neuroimaging index, increase or decrease of brain activation post-intervention, coordinates and brain region of peak voxels post-intervention, mean and standard deviation (SD) values of depressive symptoms pre-intervention, post-intervention, or difference values between them.

### Statistical Analysis

The statistical analyses mainly consisted of two parts and were performed by SD, coordinate-based ALE meta-analysis, and scale-based meta-regression.

The ALE meta-analysis was performed using Ginger ALE software (http://brainmap.org/ale/). Coordinates reported in Talairach space were first transformed into Montreal Neurological Institute (MNI) space, then MNI coordinates of all the peak voxels were divided into activation increase/decrease group and independently analyzed. For each group, the activation likelihood of each voxel was first estimated in separate studies, then the full-width at half-maximum of the Gaussian function was calculated through sample size to blur the foci, finally all the foci were combined into a whole ALE map in a random-effects model. An uncorrected *P* < 0.001 (Eickhoff *et al*., 2012) was demonstrated as threshold with a minimum cluster size of 150 mm^3^. Visualization was achieved through Mango (http://ric.uthscsa.edu/mango/).

The meta-regression was performed with the Comprehensive Meta Analysis Software (CMA: Biostat, Englewood, NJ) in random-effects model using symptomatic improvements as the study outcome. To eliminate heterogeneity between studies to the greatest extent, calculations following the Cochrane Handbook (Higgins *et al*., 2019) were preliminarily made to directly contrast depressive symptoms pre- and post-NIBS; for those who reported pre-intervention values and difference values, post-intervention values were recalculated with a recommended correlation coefficient of *r *= 0.5 (Follmann *et al*., 1992); mean and SD values of multiple-arm studies were combined into a unitary group (Supplementary Material Tables S2 and S3). For each outcome, *Z* statistics were used to test overall effect with a threshold of *P* < 0.05; standardized mean difference (SMD) and 95% confidence interval (CI) were calculated and the effect size was evaluated with Cohen's *d* (Cohen, 1992). Statistical heterogeneity was identified with *Q* statistics and quantified with *I*^2^ statistics; when *P* < 0.10, the heterogeneity was suggested as significant; when *I*^2 ^> 75%, the heterogeneity was interpreted as considerable (Higgins *et al*., 2019). Then, subgroup analysis and univariate meta-regression were used to screen sources of heterogeneity. Between-group heterogeneity during subgroup analysis was also appraised with a threshold of *P* < 0.10; the model of univariate meta-regression was assessed with *Q* statistics, and when *P* < 0.05, the covariate was indicated as a potential moderator. Finally, all the statistically significant moderators were incorporated as explanatory variables to perform a multivariate meta-regression; the *Q* statistics were delivered to detect significance of coefficients and goodness of fit; and*R*^2^ was employed to describe proportion of total between-study variance explained by the model. Publication bias was evaluated through funnel plot and Egger's test (Egger *et al*., 1997) during meta-regression to have a rough estimation of the whole study.

## Results

### Study Selection

**Figure 1:**
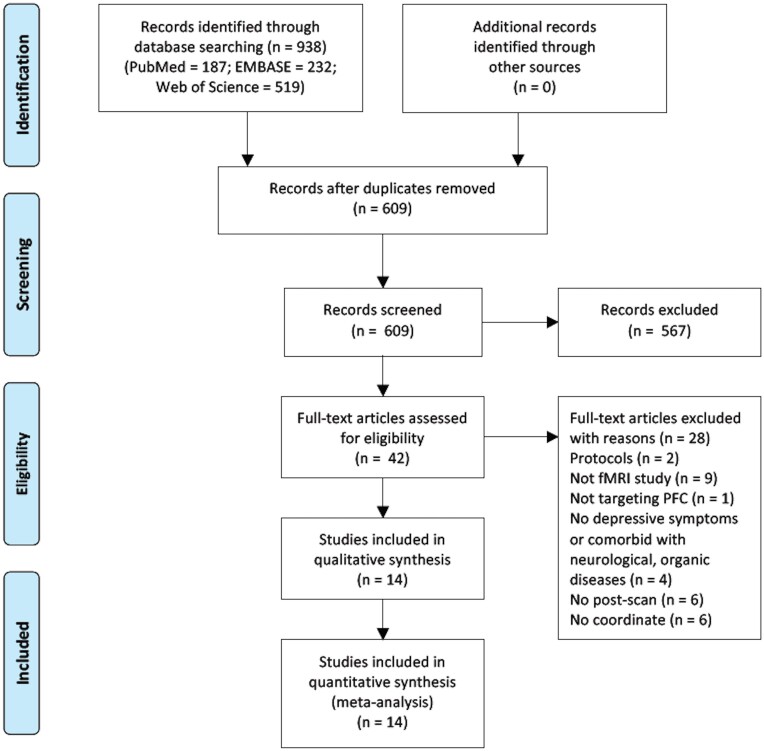
Flow diagram following the Preferred Reporting Items for Systematic Reviews and Meta-Analyses guideline.

Up to November 2020, 938 studies were identified through database searching (PubMed 187, Embase 232, Web of Science 519); after removing duplications (duplication 329), 609 studies were screened by titles and abstracts, and 567 studies were excluded; 42 studies were then assessed with full-text for eligibility and 28 studies were omitted for meeting exclusion criteria; 14 studies were incorporated in qualitative synthesis and 14 studies were finally included in our meta-analysis (Fig. 1).

### Study Characteristics

In all, 14 studies (Abend *et al*., 2019; Baeken *et al*., 2014, 2017; Chen *et al*., 2020; Eshel *et al*., 2020; Fitzgerald *et al*., 2007; Ge *et al*., 2020; Kang *et al*., 2016; Liston *et al*., 2014; Nord *et al*., 2019; Persson *et al*., 2020; Philip *et al*., 2018; Taylor *et al*., 2018; Zheng *et al*., 2020) were included in our coordinate-based ALE meta-analysis with a total sample size of 584 (Table 1), in which 11 studies reported eligible data of depressive symptoms pre- and post-intervention and were included in the scale-based meta regression. See the full description in the Supplementary Material.

**Table 1:** Characteristics of studies included in the ALE meta-analysis.

Author	Country	Year	Size	Mean Age	Type of NIBS	Original Imaging Analysis	Experimental Group	Baseline Depression Score	Size	Control Group	Baseline Depression Score	Size	Scale
Abend *et al*.	Israel	2018	19	24.7	tDCS	NA	active	NA	16	sham	NA	16	BDI
Baeken *et al*.	Belgium	2014	20	48.8	rTMS	FC	responder	25.0	7	non-responder	26.0	13	Ham-D
Baeken *et al*.	Belgium	2017	50	40.4	TBS	FC	responder	21.8	18	non-responder	21.2	26	Ham-D
Chen *et al*.	China	2020	60	46.3	rTMS	FC	active	27.0	20	sham	25.5	20	Ham-D
Eshel *et al*.	USA	2020	64	37.2	rTMS	FC	active	25.8	16	sham	25.9	12	Ham-D
Fitzgerald *et al*.	Australia	2007	26	41.2	rTMS	NA	active-HFL	34.5	15	active-LFR	33.3	11	MADRS
Ge *et al*.	Canada	2019	74	43.7	rTMS	FC	responder	22.0	29	non-responder	21.7	21	Ham-D
Kang *et al*.	Korea	2016	24	46.8	rTMS	FC	active	24.1	12	sham	20.0	9	Ham-D
Liston *et al*.	USA	2014	52	38.1	rTMS	FC	pre	25.8	17	post	16.7	17	Ham-D
Nord *et al*.	UK	2019	39	33.4	tDCS	NA	active	22.0	20	sham	21.1	19	Ham-D
Persson *et al*.	Sweden	2020	30	28.9	TBS	FC	active	29.7	11	sham	28.0	12	MADRS
Philip *et al*.	USA	2018	40	51.6	rTMS	FC	pre	47.8	35	post	30.9	35	IDSSR
Taylor *et al*.	USA	2018	32	45.5	rTMS	FC	active	25.4	16	sham	21.9	16	Ham-D
Zheng *et al*.	China	2020	54	41.1	rTMS	ALFF;FCD	pre	23.9	15	post	14.7	15	Ham-D

NA: not applicable; ALFF: amplitude of low-frequency fluctuation; FCD: functional connection degree; HFL: high-frequency left; LFR: low-frequency right; BDI: Beck Depression Inventory; Ham-D: Hamilton Rating Scale for Depression; MADRS: Montgomery-Asberg Depression Rating Scale; IDSSR: Inventory of Depressive Symptomatology, Self-Report

### Risk of Bias

**Figure 2:**
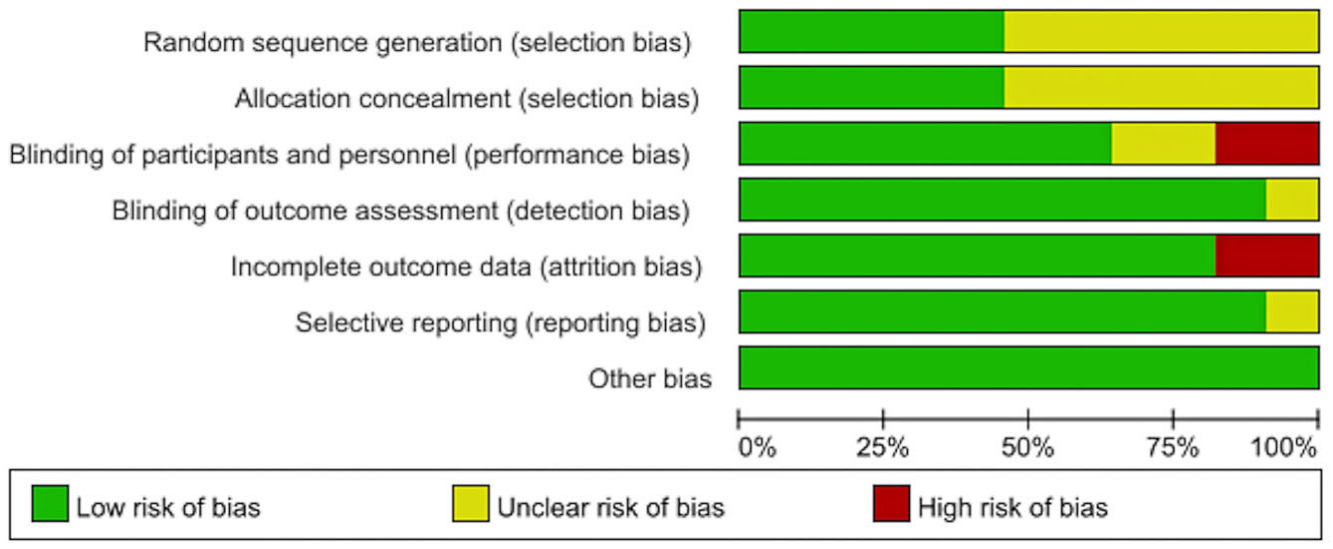
RoB graph containing items presented as percentages across all included studies.

For 11 RCTs, the RoB is summarized in Fig. 2 and Supplementary Material Fig. S1. Overall, the selection bias was mostly detected: six studies showed an unclear risk of random sequence generation and allocation concealment. Performance bias was also discovered due to the risk while blinding participants and personnel. Substantial attrition bias was caused by incomplete outcome data, considering that two studies preset ROIs rather than reporting the whole-brain coordinates. Low detection bias could be attributed to neuroimaging studies’ semi-automated operating characteristics. Low reporting bias indicated the results to be trustworthy. No other bias was found. For two cohort studies and one case-control study, an average 8.3 points’ score (9 points in total) suggested the RoB to be low. The bias mainly resulted from incomplete coordinate report (Supplementary Material Table S4). In brief, the included studies showed substantial RoB, which could be attributed to unclear randomization, imperfect blinding, and incomplete outcome data.

### Quality of Evidence

The quality assessment was composed of two parts: sample characteristics (10 points in all) and methodology and reporting (8 points in all). The first part gained an average score of 9.2 pointing, thus suggesting high quality of sample characteristics. Lowered quality was mainly due to a lack of control group; what is more, a small sample size also contributed to the downgrade during assessment. Meanwhile, average score of the second part was 7.6; several studies did not clearly report the length of the scan and no other issue was found, which indicated high methodology and reporting quality. Over all, despite the potential RoB of included studies, the reported results were generally of high quality (Supplementary Material Table S5).

### Publication Bias

**Figure 3:**
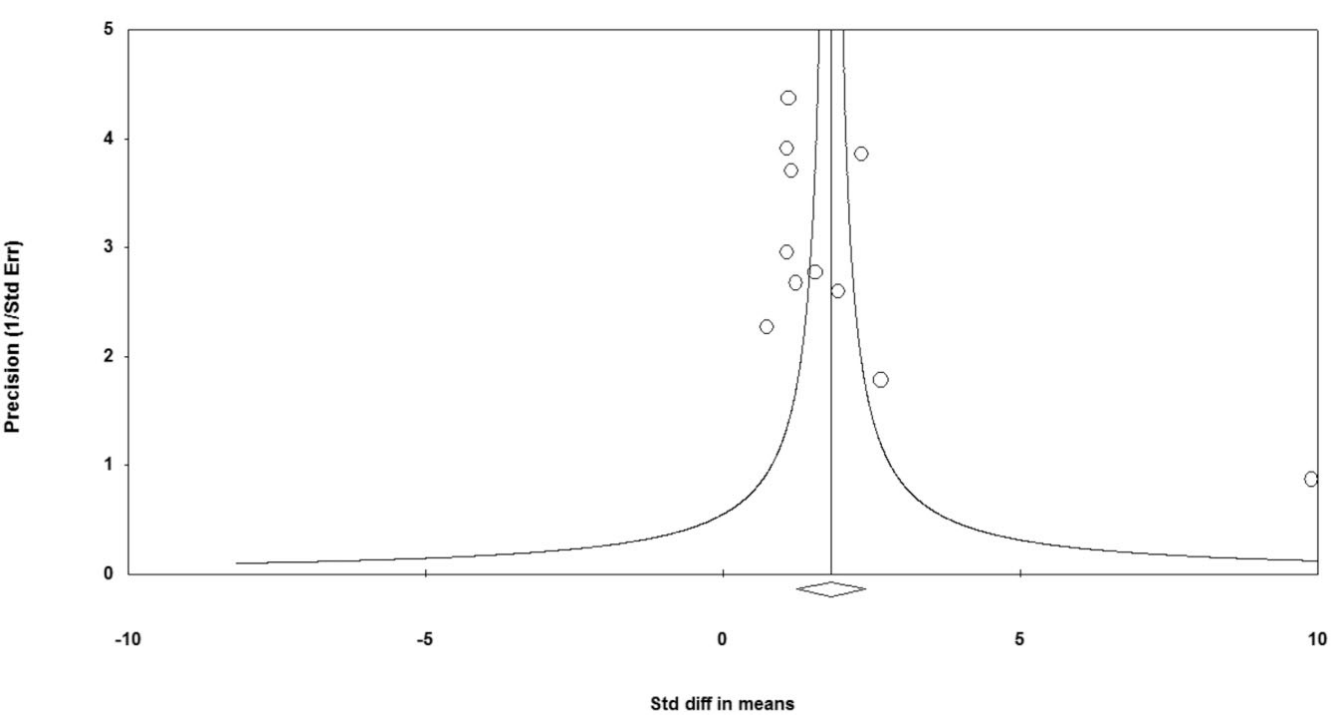
Funnel plot of precision by standard difference in means.

The asymmetry funnel plot implied substantial publication bias of included studies (Fig. 3). Egger's test further proved it (*t* = 2.57, *P* = 0.03). It is worth noting that publication bias in ALE meta-analysis is common due to its property of spatially converging effects across studies that already showed significant results (Rottschy *et al*., 2012). We minimized the risk by reporting study characteristics transparently (Müller *et al*., 2018).

### Primary Outcome

#### Coordinate-based Analysis

**Figure 4:**
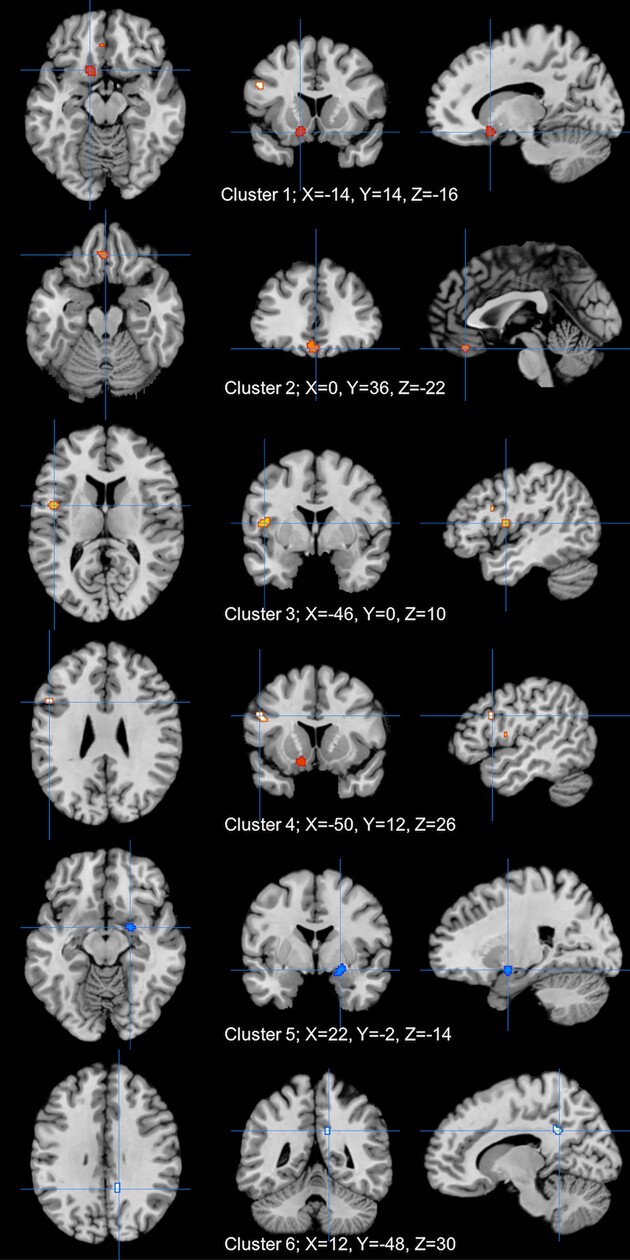
Significant alterations of brain activity caused by NIBS; clusters 1–4 (red) show activation; clusters 5 and 6 (blue) show deactivation.

Fourteen studies were included in the ALE analysis to evaluate the brain activation after NIBS. Four clusters revealed significant (*P* < 0.001) increase of activation; clusters were all located in left cerebrum, which consisted of the putamen (cluster 1, 304 mm^3^), orbitofrontal cortex and dorsal anterior cingulate cortex (cluster 2, 288 m^3^), insular cortex (cluster 3, 256 mm^3^), and dlPFC (cluster 4, 160 mm^3^). Two clusters revealed significant (*P* < 0.001) decrease of activation; clusters were all located in right cerebrum, which consisted of putamen and lateral globus pallidus and posterior entorhinal cortex (cluster 5, 224 m^3^) and dorsal posterior cingular cortex (cluster 6, 168 mm^3^) (Table 2; Fig. 4).

**Table 2:** Significant alterations of brain activations caused by NIBS.

Cluster	Side	Brain Region	*X*	*Y*	*Z*	ALE value (10^−3^)	*Z* value	Volume (mm^3^)
**Increase**								
1	Left	putamen	−14	14	−16	12.22	4.09	304
2	Left	orbitofrontal cortex	0	36	−22	10.37	3.78	288
		dorsal anterior cingulate cortex	−4	36	−18	10.31	3.77	
3	Left	insular cortex	−46	0	10	11.62	3.99	256
4	Left	dlPFC	−50	12	26	9.95	3.71	160
**Decrease**								
5	Right	putamen	22	−2	−14	10.37	3.82	224
		lateral globus pallidus						
		posterior entorhinal cortex						
6	Right	dorsal posterior cingular cortex	12	−48	30	9.85	3.73	168

Increase and decrease separately represented activate and deactivated clusters post-NIBS based on ALE meta-analysis (*P* < 0.001); *X, Y*, and *Z* represent the peak MNI coordinates, while volume represented the cluster size.

#### Scale-based Analysis

**Figure 5:**
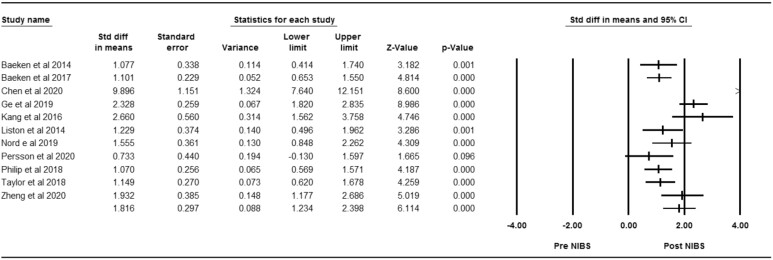
Forest plot of effectiveness concerning NIBS targeting depressive symptoms between pre- and post-intervention.

Eleven studies were included in the effect size analysis to evaluate the effectiveness of NIBS targeting depressive symptoms. Significant alterations were found between post- and pre-intervention (*Z* = 6.11, *P* < 0.01); the pooled effect size was identified as very large [*d* = 1.82, 95%CI (1.23, 2.40)]. The results indicated considerable heterogeneity (Chi squared = 75.25, *P* < 0.01; *I*^2 ^= 87%) (Fig. 5). Due to the potential publication bias caused by Chen *et al*., which showed a very significant symptomatic improvement post-NIBS, we supplemented a sensitivity analysis by omitting it and reanalyzing the rest studies; the pooled effect size remained very large [(*d* = 1.41, 95%CI (1.07, 1.76)] (Supplementary Material Fig. S2).

### Subgroup Analysis

The detailed subgroup divisions could be found in Table 3. Overall, significant between-subgroup heterogeneity was detected in the gender group (Chi squared = 3.80, *P* = 0.05; *I*^2^ = 73.70%), treatment-resistant condition group (Chi squared = 3.64, *P* = 0.06; *I*^2^ = 72.50%), comorbidity group (Chi squared = 6.53, *P* = 0.01; *I*^2^ = 84.70%), treatment duration group (Chi squared = 3.63, *P* = 0.06; *I*^2^ = 72.40%), and localization method group (Chi squared = 12.40, *P* < 0.01; *I*^2^ = 75.80%). These items were demonstrated to be significant moderators and were included in our multivariate meta-regression.

**Table 3:** Clinical moderators included in subgroup analysis and meta-regression.

	Subgroup analysis			Univariate meta-regression		
Moderator	Chi squared	*P* value	*I* ^2^ (%)	*Q* value	*P* value	Coefficient
**Study characteristics**						
Resting/task fMRI	0.33	0.57	0.00	0.09	0.77	−0.31
Sample size	1.86	0.17	46.20	0.20	0.65	−0.01
Year of publication	2.24	0.13	55.40	2.43	0.12	0.22
**Patient level**						
Age	2.18	0.14	54.20	1.28	0.26	0.05
Gender (% female)	3.80	0.05a	73.70	0.09	0.76	−0.01
**Disease level**						
Comorbidity	6.53	0.01^a^	84.70^a^	2.82	0.09	−1.13
Medication	2.70	0.10	63.00	1.20	0.27	−0.71
Treatment resistant	3.64	0.06^a^	72.50	6.65	0.01^a^	1.81
**Intervention level**						
Evaluation duration	1.90	0.17	47.50	0.35	0.55	−0.07
Intervention type	0.33	0.57	0.00	0.09	0.77	−0.31
Localization	12.40	<0.01^a^	75.80^a^	3.45	0.33	FC −0.76
						3D −1.52
						5 cm −0.22
Number of sessions	2.41	0.12	58.50	0.04	0.85	−0.01
Parameter	0.86	0.35	0.00	0.77	0.38	−0.62
Sham controlled	0.41	0.52	0.00	0.25	0.61	−0.32
Treatment duration	3.63	0.06^a^	72.40	0.11	0.74	−0.04

^a^The pooled result is statistically significant (*P* < 0.10)

### Univariate Meta-Regression

The detailed information of univariates could be found in Table 3. Overall, the significant univariate model was detected in a treatment-resistant condition group (TRD as reference group) (*Q* = 6.65, *P* = 0.01). This item was demonstrated as a significant moderator and was included in our multivariate meta-regression.

### Multivariate Meta-Regression

**Figure 6:**
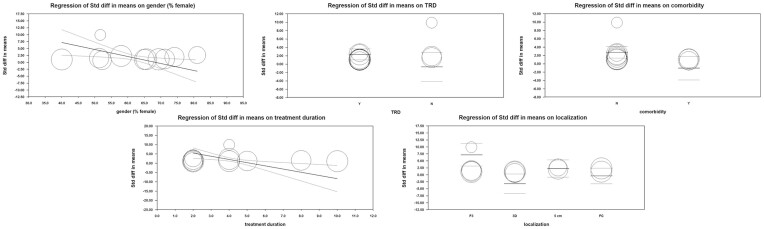
Scatterplot of significant moderators in multivariate meta-regression model.

For the multivariate meta-regression model, continuous moderators contained gender (proportion of female) and treatment duration (weeks); categorical moderators contained the treatment-resistant condition (TRD as reference group), comorbidity (no comorbidity as reference group), and localization method (Beam F3 group as the reference group). All the moderators including gender (*r* = −0.25, *Z* = −4.80, *P* < 0.01), treatment-resistant condition (*r* = −3.01, *Z* = −2.75, *P* < 0.01), comorbidity (*r* = −3.82, *Z* = −4.00, *P* < 0.01), treatment duration (*r* = −1.71, *Z* = −5.57, *P* < 0.01), and localization method (*r* FC = −7.57, *Z* FC = −4.94, *P* < 0.01; *r* 3D = −10.36, *Z* 3D = −5.73, *P* < 0.01; *r* 5cm = −4.92, *Z* 5 cm = −3.87, *P* < 0.01) reflected significant effectiveness targeting depressive symptoms. Results of test of models (*Q* = 42.32, *P* < 0.01) and goodness of fit (*Q* = 13.49, *P* < 0.01) were both significant, meanwhile 49% of total between-study variance was explained by the model (*R*^2^ = 0.49), thus indicating a good fitness of the model overall (Fig. 6 and Supplementary Material Fig. S3).

### Secondary Outcome

**Figure 7:**
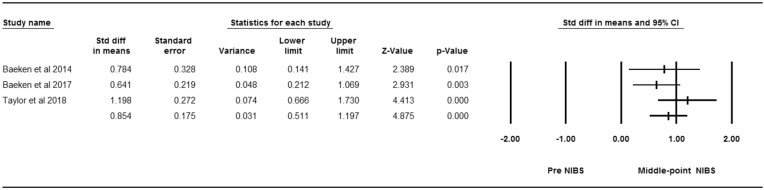
Forest plot of effectiveness concerning NIBS targeting depressive symptoms between pre- and middle-point intervention.

Three studies designed as cross-over RCTs were included in the effect size analysis to evaluate the effectiveness of NIBS targeting depressive symptoms at the middle point of intervention. Significant alterations were found between the middle point and pre- intervention (*Z* = 4.88, *P* < 0.01); the pooled effect size was identified as large [(*d* = 0.85, 95%CI (0.51, 1.20)]. The results indicated unsubstantial heterogeneity (Chi squared = 2.51, *P* = 0.28; *I*^2^ = 20%) (Fig. 7).

## Discussion

Our study delivered a combined ALE meta-analysis and meta-regression to systematically investigate effectiveness of NIBS on depressive symptoms targeting PFC in fMRI studies. In total, 14 studies with a total sample size of 584 were included in our analysis. Although substantial RoB was detected, the eligible studies were assessed as high-quality, thus suggesting our results to be reliable. Multi-dimensional improvements were detected post-intervention. On the neuroimaging level, compared to control group, both cortical and subcortical region revealed significant differences; increased activations of voxels were found in left cerebrum while decreased activations of voxels were found in right cerebrum. On the behavioral level, active-NIBS remarkably relieved depressive symptoms and the effect size was very large. What is more, several moderators showed its potential of predicting improvements post-intervention in a scale-based meta-regression model. The detailed findings are described in the following.

### NIBS balance critical depressive-related brain networks

Different brain networks are associated with own specific neural functions; deficits of neural networks both within and between them could lead to various psychiatric disorders such as depression (Ernst *et al*., 2015). Consistent with previous antidepressant studies, our results found activated central nodes of the salience network (SN; including dorsal anterior cingulate cortex and insula) and central executive network (CEN; including dlPFC) as well as deactivated central nodes of both the default mode network (DMN; including the dorsal posterior cingular cortex) and the subsystem under its control (including the posterior entorhinal cortex) after NIBS (Delaveau *et al*., 2011; Peters *et al*., 2016; Zhou *et al*., 2020). The SN, CEN, and DMN are generally regarded as core neurocognitive networks responsible for high-level cognition, among which the DMN takes charge of internally oriented cognition and is well known for its substantial relationship with MDD. CEN, on the contrary, mainly takes over the externally oriented executive attention and decision-making; reverse activity among them is supervised by the SN, an internal–external integrated network thatually facilitate thes switching between DMN and CEN (Chand *et al*., 2017; Uddin *et al*., 2011). Hyperconnectivity of DMN could be explained by consistent negative self-referential thinking in MDD, meanwhile both SN and CEN revealed anti-correlated activity with DMN; together, the imbalanced activation of the three networks leads to depressive disorders (Kaiser *et al*., 2015; Whitton *et al*., 2019). Hence, our results suggest that NIBS could relieve depressive symptoms through normalizing the dysfunctional core brain networks.

### Striatum act as a candidate biomarker in depression

Although NIBS is incapable of directly conducting deep brain stimulation, both activation and deactivation of the lentiform nucleus, which is the major component of the striatum, was discovered in our study (Brunoni *et al*., 2019).The striatum is a central reward-related brain region; deregulated corticostriatal circuits lead to anhedonia, a symptom of depression that is reflected as decreased motivation (Felger *et al*., 2016). According to our result, we hypothesize that NIBS could increase the reduced activity of the striatum and orbitofrontal cortex in the left cerebrum, both of which are critical nodes in the reward system, coupled with a compensatory deactivation of striatum in the right cerebrum (Peters *et al*., 2016; Su *et al*., 2014). Studies indicate that TMS over PFC could increase dopamine release in striatum, and higher stimulation site-striatal FC is correlated with a better treatment outcome; altogether, we further demonstrate that the potential antidepressant mechanism of NIBS on depressive symptoms might be related to the striatum on the molecular level as well as the neuroimaging level (van Schouwenburg *et al*., 2012; Avissar *et al*., 2017). Moreover, a previous study suggests that striatum could be used to predict risk for developing depression (Pan *et al*., 2017); tracing back to the source of clusters, we find that convergence of the striatum both reveals effectiveness of active-NIBS and implies a symptom-related change of FC, thus emphasizing its potential for predicting intervention outcome in future studies (Abend *et al*., 2019; Baeken *et al*., 2017; Eshel *et al*., 2020). In all, the striatum plays a central role in NIBS and could be a candidate biomarker for both diagnosis and treatment of patients with depression.

### Clinical factors indicate improvement of depressive symptoms post-NIBS

Consistent with previous studies, the results of our meta-regression indicate that moderators in patient aspect, disease aspect, and intervention aspect all contribute to the effectiveness of NIBS (Kar *et al*., 2019). For patients’ characteristics, the higher proportion of females result in a more negative treatment response. Since gender difference has been long-existant in depression, we suppose the anti-correlation could be caused by either the larger prevalence of female patients with depression or the possible risk factors that lead to this gender gap (Kuehner, 2017). On the disease level, in accordance with general considerations that NIBS is recommended in at least stage-3 MDD, the intervention shows a better effect in TRD patients (Kraus *et al*., 2020); meanwhile, patients with comorbidity react worse to NIBS. Similarly, one study concerning bipolar patients with auditory verbal hallucinations found that tDCS could relieve those hallucinations, but the depressive symptoms were not improved (Zhuo *et al*., 2020). Altogether, these findings suggest that mixed symptoms require their own symptom-specific treatment modalities, and more personalized NIBS are needed in clinical practice (Siddiqi *et al*., 2020).

As for the intervention level, the main results are as follows: first, contrary to our secondary outcome that effect size measured at the middle-point is smaller than that at the end-point, longer treatment duration brings about less effectiveness. This contradiction could be attributed to the fact that mechanism of the long-lasting effect caused by long-term potentiation remains unclear, and hence we cannot decide on an exactly appropriate duration of NIBS (Cirillo *et al*., 2017). Nevertheless, the Stanford Accelerated Intelligent Neuromodulation Therapy indicates that a short-term yet high-dose treatment shows significant efficacy for TRD compared to the general protocol, which enables us to look at the relationship between treatment duration and clinical outcome from a new perspective (Cole *et al*., 2020). Second, our results demonstrate that the F3 Beam method, which localizes the target region based on an electroencephalography 10–20 system, give rises to the best clinical outcome compared with the other three methods, even though the FC-guided localization method has been widely proven to be effective (Whitton *et al*., 2019; Cash *et al*., 2020). Other than the instability of functional-based localization that can be potentially caused by head movement, the results indicate that the sgACC-dlPFC (subgenual anterior cingulate cortex) guided approach may not be the most suitable protocol for all patients, which reemphasizes the need for the more personalized interventions mentioned before. In all, considering that various clinical characteristics could influence effectiveness of NIBS, it is of great importance to subtype patients with depression accordingly, so that more precise and individualized treatments could be delivered in the future.

Several limitations can be found in our study. First, considering the large cost of each fMRI scan, the number of studies included in our ALE meta-analysis is relatively small and may cause probable bias, due to which we are unable to contrast the neuroimaging differences resulting from various methods (TMS and tES) and target regions (dlPFC, dmPFC, vlPFC, vmPFC, etc.) (Müller *et al*., 2018). Second, most studies employed FC as a neuroimaging indicator, which required a seed region; a few studies reported coordinates of ROI rather than the whole brain; although a previous study suggested that influences generated from these preliminary settings were limited, so inevitable bias may still exist (Zhong *et al*., 2016). As such, despite the efforts we made by plotting subgroup analyses and meta-regression, the heterogeneity between included articles remain substantial. More comprehensive and homogeneous RCTs are required to better characterize neural mechanisms of NIBS targeting PFC on depression in future studies. Further, some newly published articles after our study research indicated the urgent need for more timeliness reviews with an inclusion of the latest findings in the future. Notably, despite that no replicate ALE meta-analysis regarding NIBS targeting the PFC on depression has been published to date to our knowledge, as has been recently addressed by Downar *et al*., the antidepressant effect of TMS might be attributed to the activation of the SN and deactivation of the DMN (Downar *et al*., 2024), which potentially added reliability to our study findings.

Taken together, our findings indicate NIBS targeting the PFC to be an effective neuromodulation strategy to improve depressive symptoms, meanwhile emphasizing the importance of promoting the precision and objectiveness of treatments in depression. Notably, the emergence of novel NIBS targets (i.e. the visual cortex) and techniques (i.e. the transcutaneous auricular/cervical vagus nerve stimulation) in recent years has yielded new insights into the psychiatric field, holding stronger promise for performing more targeted treatments over traditional pharmacology. With the combinations of machine learning methods, we have recently made an innovative attempt by developing a neuroimaging-based subtyping and precise rTMS strategy and implemented it in depressed youths (Xiao *et al*., 2024). Post 2-week precise rTMS, neuroimaging deficits were significantly normalized, corresponding to significant symptomatic improvements (Xiao *et al*., 2024). Combined with current findings, we further advocate an integration of psychological and biological outcomes including both neurobiological measures and psychological assessments of mood and cognition, as well as an expansion from fMRI to multimodal neuroimaging techniques such as positron emission tomography, diffusion tensor imaging, or electroencephalography, for the establishment of an objective neurobiomarker-based precision medicine framework in a diagnoses-treatment-evaluation closed-looped beyond symptoms, eventually guiding clinical practice in the psychiatric field.

## Conclusions

Significant alterations of brain activations and improvements of depressive symptoms can be found after NIBS targeting the PFC in fMRI studies. For the cortical region, deficits of important cognitive neural networks including the SN, CEN, and DMN are normalized post-intervention. For the subcortical region, the striatum plays the role of a candidate biomarker of NIBS when treating depressive symptoms. Multi-level clinical factors including gender difference, treatment-resistant condition, comorbidity, treatment duration, and method of localization reveal the potential of being employed as moderators for effectiveness of NIBS. More high-quality, multi-center RCTs delivering personalized NIBS are urged for future clinical practice.

## Supplementary Material

kkae025_Supplemental_File
