# Supplementary material for: Effectiveness of non-invasive brain stimulation on depressive symptoms targeting prefrontal cortex in functional magnetic resonance imaging studies: a combined systematic review and meta-analysis
Source: Psychoradiology. 2024 Nov 5;4:kkae025. doi: 10.1093/psyrad/kkae025 (PMC11629992; doi:10.1093/psyrad/kkae025)
Supplement: kkae025_Supplemental_File [file kkae025_supplemental_file.docx]

**Supplementary Materials**

**Study Characteristics**

In all, 14 studies were included in our coordinate-based ALE meta-analysis with a total sample size of 584; 11 studies reported eligible data of depressive symptoms pre & post intervention and were included in the scale-based meta regression.

Studies were published in Asia, Europe, North America and Oceania, ranging from 2007 to 2020. Seven studies were designed as standard RCTs, 3 studies were designed as cross-over control trails and 1 study was designed as before-after control trail, among which 9 studies were sham-controlled; besides, 2 cohort studies and 1 case-control study were also included in our analysis. Eleven studies reported coordinates of whole-brain voxels and 3 studies preset a region of interest (ROI); neuroimaging indicators contained FC, ALFF, functional connection degree (FCD) and raw BOLD signals. Three studies were task fMRI studies, and the other 11 were resting-state fMRI studies. Nine studies delivered rTMS treatment with a mixed frequency of 5-20Hz, 3 studies delivered iTBS treatment and 2 studies delivered tDCS treatment, one of which used a constant current of 1mA while the other 1.5mA. Two studies targeted at medical PFC and the rest 12 studies targeted at lateral PFC (all dlPFC); as for the localization of intervention target region, 4 studies were localized through Beam F3 method, 4 studies were localized through precise structural 3D method, 2 studies were localized through sgACC-dlPFC functional connectivity, 2 studies were localized through “5 cm” rule, while 2 studies didn’t clearly report method of localization. The number of total NIBS sessions varied from 2 to 40, following a 1-10 weeks’ treatment duration and evaluation duration. The mean age of participants revealed a relatively large span from 24.7 to 51.6, with an average proportion of 58% female. Four studies employed a less strict inclusion criteria and participants comorbid with bipolar disorder, post-traumatic stress disorder (PTSD), anxiety were included; 9 studies restricted participants to be diagnosed with MDD without comorbidity; 1 study recruited healthy volunteers with depressive symptoms. Participants of 10 studies suffered from treatment-resistant depression; participants of 7 studies either experienced a medication wash-out in advance or remained medication-naive, and hence were medication-free, meanwhile participants of the other 7 studies were medicated during NIBS.

**Supplementary Material Table S1:** Key words for systematic search

| **Database** | **Index and Key Words** |
| --- | --- |
| PubMed | "noninvasive brain stimulation"[Title/Abstract] OR "NIBS"[Title/Abstract] OR "transcranial direct current stimulation"[MeSH Terms] OR "transcranial electrical stimulation"[Title/Abstract] OR "repetitive transcranial electrical stimulation"[Title/Abstract] OR "electric stimulation therapy"[Title/Abstract] OR "transcutaneous electric nerve stimulation"[Title/Abstract] OR "tES"[Title/Abstract] OR "transcranial direct current stimulation"[Title/Abstract] OR "tDCS"[Title/Abstract] OR "transcranial alternating current stimulation"[Title/Abstract] OR "tACS"[Title/Abstract] OR "transcranial random noise stimulation"[Title/Abstract] OR "tRNS"[Title/Abstract] OR "transcranial magnetic stimulation"[MeSH Terms] OR "transcranial magnetic stimulation"[Title/Abstract] OR "TMS"[Title/Abstract] OR "repetitive transcranial magnetic stimulation"[Title/Abstract] OR "rTMS"[Title/Abstract] OR "theta burst stimulation"[Title/Abstract] OR "TBS"[Title/Abstract]  **AND**  "depressive disorder"[MeSH Terms] OR "depression"[MeSH Terms] OR "major depressive disorder"[Title/Abstract] OR "depression"[Title/Abstract] OR "depressive"[Title/Abstract] OR "depressed"[Title/Abstract]  **AND**  "prefrontal cortex"[MeSH Terms] OR "dorsolateral"[Title/Abstract] OR "dorsomedial"[Title/Abstract] OR "ventrolateral"[Title/Abstract] OR "ventromedial"[Title/Abstract] OR "cortex"[Title/Abstract] OR "dorsal"[Title/Abstract] OR "ventral"[Title/Abstract] OR "lateral"[Title/Abstract] OR "medial"[Title/Abstract] OR "dlpfc"[Title/Abstract] OR "dmpfc"[Title/Abstract] OR "vmpfc"[Title/Abstract] OR "vlpfc"[Title/Abstract] OR "PFC"[Title/Abstract] OR "prefrontal cortex"[Title/Abstract]  **AND**  "magnetic resonance imaging"[MeSH Terms] OR "magnetic resonance imaging"[Title/Abstract] OR "MRI"[Title/Abstract] OR "functional magnetic resonance imaging"[Title/Abstract] OR "functional mri"[Title/Abstract] OR "fMRI"[Title/Abstract] |
| EMBASE | 'noninvasive brain stimulation':ab,ti OR nibs:ab,ti OR 'electric stimulation therapy':ab,ti OR 'transcutaneous electric nerve stimulation':ab,ti OR 'repetitive transcranial electrical stimulation':ab,ti OR 'transcranial electrical stimulation':ab,ti OR tes:ab,ti OR 'transcranial direct current stimulation':ab,ti OR tdcs:ab,ti OR 'transcranial alternating current stimulation':ab,ti OR tacs:ab,ti OR 'transcranial random noise stimulation':ab,ti OR trns:ab,ti OR 'transcranial magnetic stimulation':ab,ti OR tms:ab,ti OR 'repetitive transcranial magnetic stimulation':ab,ti OR rtms:ab,ti OR 'theta burst stimulation':ab,ti OR tbs:ab,ti  **AND**  'major depressive disorder':ab,ti OR depression:ab,ti OR depressive:ab,ti OR depressed:ab,ti  **AND**  dorsolateral:ab,ti OR dorsomedial:ab,ti OR ventrolateral:ab,ti OR ventromedial:ab,ti OR cortex:ab,ti OR dorsal:ab,ti OR ventral:ab,ti OR lateral:ab,ti OR medial:ab,ti OR dlpfc:ab,ti OR dmpfc:ab,ti OR vmpfc:ab,ti OR vlpfc:ab,ti OR pfc:ab,ti OR 'prefrontal cortex':ab,ti  **AND**  'functional magnetic resonance imaging':ab,ti OR 'functional mri':ab,ti OR fmri:ab,ti |
| Web of Science | TOPIC:(noninvasive brain stimulation) ORTOPIC: (NIBS) OR TOPIC: (electric stimulation therapy) OR TOPIC:(transcutaneous electric nerve stimulation) OR TOPIC: (repetitive transcranial electrical stimulation) ORTOPIC: (transcranial electrical stimulation) OR TOPIC: (tES) ORTOPIC: (transcranial direct current stimulation) OR TOPIC: (tDCS) ORTOPIC: (transcranial alternating current stimulation) OR TOPIC: (tACS) OR TOPIC: (transcranial random noise stimulation) OR TOPIC: (tRNS) ORTOPIC: (transcranial magnetic stimulation) OR TOPIC: (TMS) ORTOPIC: (repetitive transcranial magnetic stimulation) OR TOPIC:(rTMS) OR TOPIC: (theta burst stimulation) OR TOPIC: (TBS)  **AND**  TOPIC: (major depressive disorder) OR TOPIC:(depression) OR TOPIC: (depressive) OR TOPIC: (depressed)  **AND**  TOPIC: (prefrontal cortex) OR TOPIC: (dorsolateral) OR TOPIC: (dorsomedial) OR TOPIC: (ventrolateral) OR TOPIC: (ventromedial) OR TOPIC: (cortex) OR TOPIC: (dorsal) OR TOPIC: (ventral) OR TOPIC: (lateral) OR TOPIC: (medial) OR TOPIC: (dlpfc) OR TOPIC: (dmpfc) OR TOPIC: (vmpfc) OR TOPIC: (vlpfc) OR TOPIC: (PFC) OR TOPIC: (prefrontal cortex)  **AND**  (functional magnetic resonance imaging) OR TOPIC: (functional mri) OR TOPIC: (fMRI) |

**Supplementary Material Table S2:** Formulae for group combinations

|  | Group 1 | Group 2 | Combined Group |
| --- | --- | --- | --- |
| Sample Size | $N_{1}$ | $N_{2}$ | $N_{1}{+N}_{2}$ |
| Mean Score | $M_{1}$ | $M_{2}$ | $\frac{N_{1}M_{1}+N_{2}M_{2}}{N_{1}{+N}_{2}}$ |
| Standard Deviation | $SD_{1}$ | $SD_{2}$ | $\sqrt{\frac{\left( N_{1}-1 \right){SD_{1}}^{2}+{(N}_{2}-1){SD_{2}}^{2}+\frac{{N_{1}N}_{2}}{N_{1}{+N}_{2}}\left( {M_{1}}^{2}+{M_{2}}^{2}-2M_{1}M_{2} \right)}{N_{1}{+N}_{2}-1}}$ |

**Supplementary Material Table S3:** Formulae for calculating final mean, standard deviation (SD) scores using baseline scores and changes

|  | Baseline | Change | Final |
| --- | --- | --- | --- |
| Group 1 ($N$) | ${Mean}_{baseline}\pm{SD}_{baseline}$ | ${Mean}_{change}\pm{SD}_{change}$ | ${Mean}_{final}\pm{SD}_{final}$ |
| ${Mean}_{final}$ | $\mathrm{Mean}_{\mathrm{baseline}}-{Mean}_{change}$ | | |
| ${SD}_{final}$ | $\sqrt{{{SD}_{baseline}}^{2}+{{SD}_{change}}^{2}-2\times r\times{SD}_{baseline}\times{SD}_{change}}$ | | |

**Supplementary Material Table S4:** Scores of Newcastle-Ottawa Quality Assessment Scale

| **Author** | **Selection** | | | | **Comparability** | **Outcome/Exposure** | | |
| --- | --- | --- | --- | --- | --- | --- | --- | --- |
| **Cohort Study** | Selection 1 | Selection 2 | Selection 3 | Selection 4 | Comparability | Outcome 1 | Outcome 2 | Outcome 3 |
| Liston et al 2014 [1] | 1 | 1 | 1 | 1 | 2 | 0 | 1 | 1 |
| Zheng et al 2020 [2] | 1 | 1 | 1 | 1 | 2 | 1 | 1 | 1 |
| **Case-control Study** | Selection 1 | Selection 2 | Selection 3 | Selection 4 | Comparability | Exposure 1 | Exposure 2 | Exposure 3 |
| Ge et al 2019 [3] | 1 | 1 | 0 | 1 | 2 | 1 | 1 | 1 |

**Supplementary Material Table S5:** Quality assessment

| **Author** | **Category 1: Sample Characteristics** | | | | | **Category 2: Methodology and Reporting** | | | | | | | |
| --- | --- | --- | --- | --- | --- | --- | --- | --- | --- | --- | --- | --- | --- |
|  | **No.1** | **No.2** | **No.3** | **No.4** | **No.5** | **No.6** | **No.7** | **No.8** | **No.9** | **No.10** | **No.11** | **No.12** | **No.13** |
| Abend et al 2018 [4] | 0 | 2 | 0 | 1 | 2 | 1 | 1 | 1 | 1 | 1 | 1 | 1 | 1 |
| Baeken et al 2014 [5] | 1 | 2 | 1 | 3 | 0 | 1 | 1 | 1 | 1 | 1 | 1 | 1 | 1 |
| Baeken et al 2017 [6] | 1 | 2 | 2 | 3 | 2 | 1 | 1 | 1 | 1 | 1 | 1 | 1 | 1 |
| Chen et al 2020 [7] | 1 | 2 | 2 | 3 | 2 | 1 | 0 | 1 | 1 | 1 | 1 | 1 | 1 |
| Eshel et al 2020 [8] | 1 | 2 | 2 | 3 | 2 | 1 | 1 | 1 | 1 | 1 | 1 | 1 | 1 |
| Fitzgerald et al 2007 [9] | 1 | 2 | 2 | 3 | 2 | 1 | 1 | 1 | 1 | 1 | 1 | 1 | 1 |
| Ge et al 2019 [3] | 1 | 2 | 2 | 3 | 2 | 1 | 0 | 1 | 1 | 1 | 1 | 1 | 1 |
| Kang et al 2016 [10] | 1 | 2 | 2 | 3 | 1 | 1 | 0 | 1 | 1 | 1 | 1 | 1 | 1 |
| Liston et al 2014 [1] | 1 | 2 | 2 | 3 | 2 | 1 | 0 | 1 | 1 | 1 | 1 | 1 | 1 |
| Nord e al 2019 [11] | 1 | 2 | 2 | 3 | 2 | 1 | 1 | 1 | 1 | 1 | 1 | 1 | 1 |
| Persson et al 2020 [12] | 1 | 2 | 2 | 3 | 2 | 1 | 1 | 1 | 1 | 1 | 1 | 1 | 1 |
| Philip et al 2018 [13] | 1 | 2 | 0 | 3 | 2 | 1 | 1 | 1 | 1 | 1 | 1 | 1 | 1 |
| Taylor et al 2018 [14] | 1 | 2 | 2 | 3 | 2 | 1 | 0 | 1 | 1 | 1 | 1 | 1 | 1 |
| Zheng et al 2020 [2] | 1 | 2 | 2 | 3 | 2 | 1 | 1 | 1 | 1 | 1 | 1 | 1 | 1 |

**
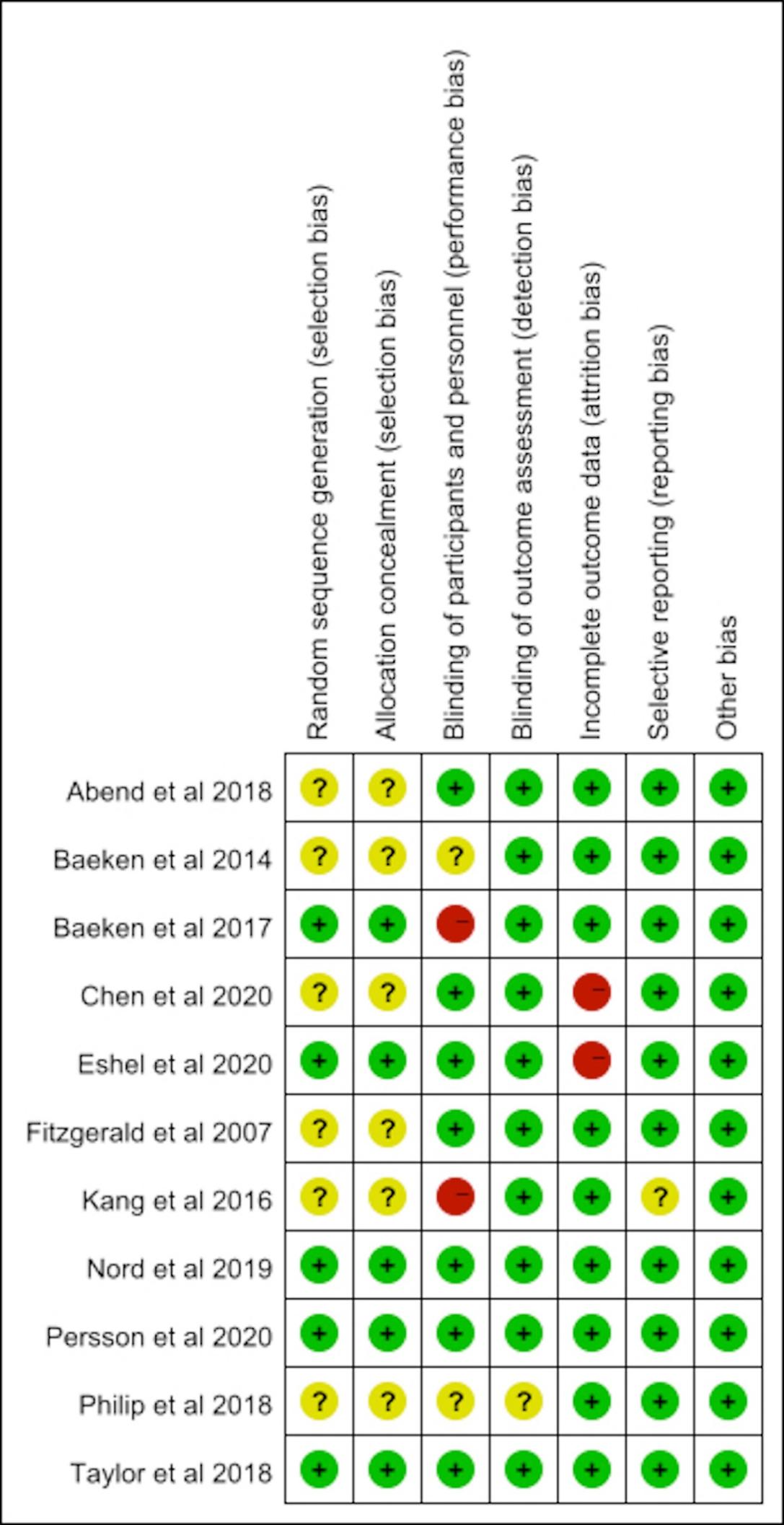
**

**Supplementary Material Figure S1:** Risk of bias graph containing items for each included study

**
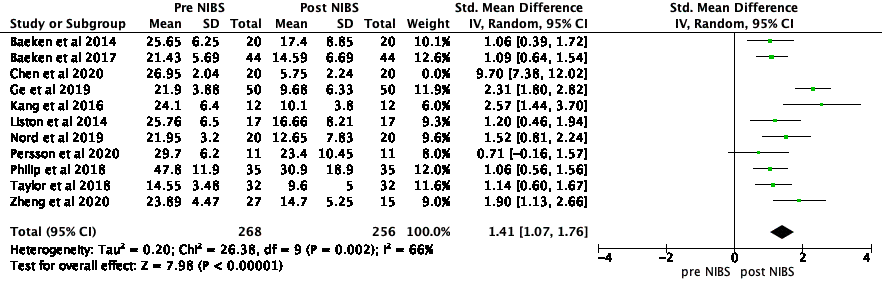
**

**Supplementary Material Figure S2:** Forest plot of effectiveness concerning non-invasive brain stimulation targeting depressive symptoms between pre & post intervention after omitting Chen et al. 2020

**
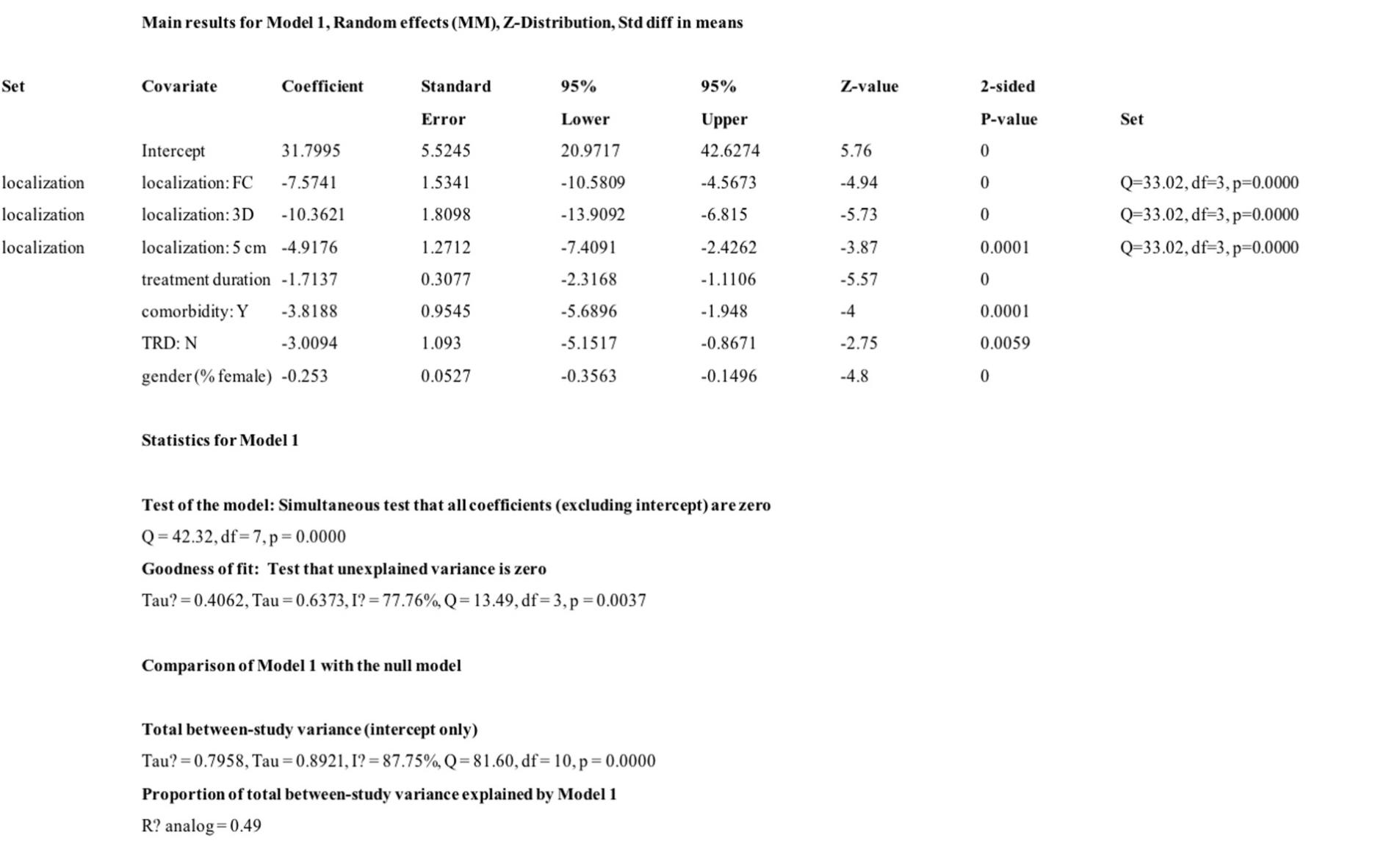
**

**Supplementary Material Figure S3:** Multivariate meta-regression model

**Reference**

1. Liston C, Chen AC, Zebley BD, Drysdale AT, Gordon R, Leuchter B, Voss HU, Casey BJ, Etkin A, Dubin MJ. Default mode network mechanisms of transcranial magnetic stimulation in depression. Biol Psychiatry. 2014 Oct 1;76(7):517-26. doi: 10.1016/j.biopsych.2014.01.023.
2. Zheng A, Yu R, Du W, Liu H, Zhang Z, Xu Z, Xiang Y, Du L. Two-week rTMS-induced neuroimaging changes measured with fMRI in depression. J Affect Disord. 2020 Jun 1;270:15-21. doi: 10.1016/j.jad.2020.03.038.
3. Ge R, Downar J, Blumberger DM, Daskalakis ZJ, Vila-Rodriguez F. Functional connectivity of the anterior cingulate cortex predicts treatment outcome for rTMS in treatment-resistant depression at 3-month follow-up. Brain Stimul. 2020 Jan-Feb;13(1):206-214. doi: 10.1016/j.brs.2019.10.012.
4. Abend R, Sar-El R, Gonen T, Jalon I, Vaisvaser S, Bar-Haim Y, Hendler T. Modulating Emotional Experience Using Electrical Stimulation of the Medial-Prefrontal Cortex: A Preliminary tDCS-fMRI Study. Neuromodulation. 2019 Dec;22(8):884-893. doi: 10.1111/ner.12787.
5. Baeken C, Marinazzo D, Wu GR, Van Schuerbeek P, De Mey J, Marchetti I, Vanderhasselt MA, Remue J, Luypaert R, De Raedt R. Accelerated HF-rTMS in treatment-resistant unipolar depression: Insights from subgenual anterior cingulate functional connectivity. World J Biol Psychiatry. 2014 May;15(4):286-97. doi: 10.3109/15622975.2013.872295.
6. Baeken C, Duprat R, Wu GR, De Raedt R, van Heeringen K. Subgenual Anterior Cingulate-Medial Orbitofrontal Functional Connectivity in Medication-Resistant Major Depression: A Neurobiological Marker for Accelerated Intermittent Theta Burst Stimulation Treatment? Biol Psychiatry Cogn Neurosci Neuroimaging. 2017 Oct;2(7):556-565. doi: 10.1016/j.bpsc.2017.01.001.
7. Chen FJ, Gu CZ, Zhai N, Duan HF, Zhai AL, Zhang X. Repetitive Transcranial Magnetic Stimulation Improves Amygdale Functional Connectivity in Major Depressive Disorder. Front Psychiatry. 2020 Jul 31;11:732. doi: 10.3389/fpsyt.2020.00732.
8. Eshel N, Keller CJ, Wu W, Jiang J, Mills-Finnerty C, Huemer J, Wright R, Fonzo GA, Ichikawa N, Carreon D, Wong M, Yee A, Shpigel E, Guo Y, McTeague L, Maron-Katz A, Etkin A. Global connectivity and local excitability changes underlie antidepressant effects of repetitive transcranial magnetic stimulation. Neuropsychopharmacology. 2020 May;45(6):1018-1025. doi: 10.1038/s41386-020-0633-z.
9. Fitzgerald PB, Sritharan A, Daskalakis ZJ, de Castella AR, Kulkarni J, Egan G. A functional magnetic resonance imaging study of the effects of low frequency right prefrontal transcranial magnetic stimulation in depression. J Clin Psychopharmacol. 2007 Oct;27(5):488-92. doi: 10.1097/jcp.0b013e318151521c.
10. Kang JI, Lee H, Jhung K, Kim KR, An SK, Yoon KJ, Kim SI, Namkoong K, Lee E. Frontostriatal Connectivity Changes in Major Depressive Disorder After Repetitive Transcranial Magnetic Stimulation: A Randomized Sham-Controlled Study. J Clin Psychiatry. 2016 Sep;77(9):e1137-e1143. doi: 10.4088/JCP.15m10110.
11. Nord CL, Halahakoon DC, Limbachya T, Charpentier C, Lally N, Walsh V, Leibowitz J, Pilling S, Roiser JP. Neural predictors of treatment response to brain stimulation and psychological therapy in depression: a double-blind randomized controlled trial. Neuropsychopharmacology. 2019 Aug;44(9):1613-1622. doi: 10.1038/s41386-019-0401-0.
12. Persson J, Struckmann W, Gingnell M, Fällmar D, Bodén R. Intermittent theta burst stimulation over the dorsomedial prefrontal cortex modulates resting-state connectivity in depressive patients: A sham-controlled study. Behav Brain Res. 2020 Sep 15;394:112834. doi: 10.1016/j.bbr.2020.112834.
13. Philip NS, Barredo J, van 't Wout-Frank M, Tyrka AR, Price LH, Carpenter LL. Network Mechanisms of Clinical Response to Transcranial Magnetic Stimulation in Posttraumatic Stress Disorder and Major Depressive Disorder. Biol Psychiatry. 2018 Feb 1;83(3):263-272. doi: 10.1016/j.biopsych.2017.07.021.
14. Taylor SF, Ho SS, Abagis T, Angstadt M, Maixner DF, Welsh RC, Hernandez-Garcia L. Changes in brain connectivity during a sham-controlled, transcranial magnetic stimulation trial for depression. J Affect Disord. 2018 May;232:143-151. doi: 10.1016/j.jad.2018.02.019.
